# A 24-Month Prospective Study of the Effects of Sleeve Gastrectomy on Glucose Homeostasis in Youth

**DOI:** 10.3390/nu18050734

**Published:** 2026-02-25

**Authors:** Ana Paola Lopez Lopez, Imen Becetti, Meghan Lauze, Karen Olivar Carreno, Hang Lee, Vibha Singhal, Miriam A. Bredella, Madhusmita Misra

**Affiliations:** 1 Division of Pediatric Endocrinology, Mass General for Children, Harvard Medical School, Boston, MA 02114, USA; alopezlopez@mgh.harvard.edu (A.P.L.L.); ibecetti@mgh.harvard.edu (I.B.); vibhasinghal@mednet.ucla.edu (V.S.); 2 Neuroendocrine Unit, Massachusetts General Hospital, Harvard Medical School, Boston, MA 02114, USA; meghan.lauze@umassmed.edu (M.L.); karen.olivarc@gmail.com (K.O.C.); 3 Biostatistics Center, Massachusetts General Hospital, Boston, MA 02114, USA; hlee5@mgh.harvard.edu; 4 Department of Pediatrics, Division of Endocrinology, David Geffen School of Medicine, University of California Los Angeles, Los Angeles, CA 90095, USA; 5 Department of Radiology, Musculoskeletal Imaging and Interventions, Massachusetts General Hospital, Harvard Medical School, Boston, MA 02114, USA; miriam.bredella@nyulangone.org; 6 Department of Radiology, NYU Langone Health, New York, NY 10016, USA; 7 Department of Pediatrics, University of Virginia, Charlottesville, VA 22903, USA

**Keywords:** glucose homeostasis, sleeve gastrectomy, obesity, youth

## Abstract

**Background:** Metabolic and bariatric surgery (MBS) results in significant changes in weight and body composition, along with glucose homeostasis improvement and type 2 diabetes resolution. In youth, sleeve gastrectomy (SG) is the most frequently performed MBS. Long-term studies assessing the duration over which glucose homeostasis parameters improve after SG are sparse. **Objective:** To examine the duration over which SG impacts glucose homeostasis in youth. **Methods:** This 24-month prospective study enrolled 65 youth (49 females) with moderate–severe obesity (mean age 18.0 ± 0.3 years). A total of 28 underwent SG, while 37 non-surgical (NS) participants received routine lifestyle counseling. At baseline, 12- and 24-month follow-up visits, HbA1c, and fasting and post-prandial insulin and glucose levels at 15, 30, 60, 90, and 120 min following a mixed meal tolerance test were obtained, and body composition was assessed. **Results:** At baseline, SG vs. NS had higher weight, body mass index (BMI) and percent fat mass (*p* ≤ 0.019), but did not differ for glucose homeostasis parameters. Over 24 months, reductions in weight-related parameters were noted in SG vs. NS (*p* ≤ 0.0001). Over 12 and 24 months, improvements occurred in HbA1c, fasting insulin, Homeostatic Model Assessment for Insulin Resistance (HOMA-IR), and the Matsuda index in SG vs. NS (*p* ≤ 0.002). However, the between-group difference for changes in glucose homeostasis parameters between 12 and 24 months was not significant. **Conclusions:** Improvements in glucose homeostasis occur mostly in the first year following SG, with subsequent stabilization of these measures.

## 1. Introduction

Childhood obesity rates have been rapidly rising, affecting approximately 14.7 million U.S children between 2017 and 2020 [1]. The early onset of obesity in youth has been accompanied by an increased risk of chronic obesity-related metabolic complications, including prediabetes, type 2 diabetes mellitus (T2D), and cardiovascular disease, resulting in poor health outcomes, long-term disability, and increased healthcare costs [2,3,4].

Metabolic and bariatric surgery (MBS), including sleeve gastrectomy (SG), is one of the most effective treatment modalities for youth with obesity, providing persistent weight loss along with improvement or remission of obesity-related comorbidities [5,6]. In particular, many patients undergoing MBS experience resolution of T2D, with one study in adults demonstrating long-term reduction in the incidence of T2D 20 years following MBS [7,8,9]. Similar data have been published in youth, with the Teen-LABS Consortium reporting that 95% of patients achieved resolution of T2D within three years of either Roux-en-Y gastric bypass (RYGB) or SG with 76% of patients having resolution of prediabetes [6]. We reported remission of T2D in 87.5% of adolescents vs. 54.8% of adults undergoing gastric bypass or sleeve gastrectomy over four years [10]. Similarly, five years after MBS, Inge et al. demonstrated that adolescents receiving gastric bypass were more likely to achieve T2D remission compared to their adult counterparts [5]. Recently, a study by de la Cruz-Muñoz et al. reported complete remission in five adolescents with pre-existing T2D/hyperglycemia 10–18 years following MBS [11]. Such promising data have positioned MBS as a powerful yet underutilized therapeutic option for youth with obesity and T2D [12], with greater efficacy than medical therapy [13].

Despite the many studies reporting overall remission rates of T2D and prediabetes in adolescents post-MBS, data are lacking on how glycemic control is altered over time following MBS. Here, we report changes in glucose homeostasis parameters in youth with obesity over 24 months following SG, with a particular focus on whether further improvements in insulin resistance (IR) and beta cell function (BCF) markers are evident between 12 and 24 months after SG. We have previously demonstrated that our cohort experienced most of the weight loss in the first 12 months post-SG, which was sustained at a 24-month follow-up, with a plateauing of weight between 12 and 24 months [14,15]. Based on the observed weight trajectory, we hypothesized that youth with obesity undergoing SG would experience an overall improvement in glycemic control over 24 months, with the greatest effects seen in the first 12 months post SG consistent with their weight loss pattern.

## 2. Materials and Methods

Study Design: In this 24-month longitudinal observational study, 65 adolescents and young adults (49 females) were enrolled according to the following inclusion criteria: (i) age 13–25 years and (ii) severe obesity to qualify for MBS, defined as BMI of ≥35 kg/m^2^ or ≥120% of the 95th percentile BMI for sex and age with the presence of at least one obesity-related complication, or a BMI ≥ 40 kg/m^2^ or ≥140% of the 95th percentile BMI [16]. Twenty-eight participants underwent SG, and 37 participants received routine lifestyle counseling and were non-surgical (NS) controls. Choice for surgery vs. non-surgical lifestyle management was determined by the participant, family, and clinical providers prior to study enrollment. Exclusion criteria included disorders or medications with potential effects on bone metabolism (except vitamin D and/or calcium supplementation or use of hormonal contraceptives), substance abuse per DSM-5 criteria, smoking ≥ ten cigarettes/day, pregnancy, or breastfeeding. Study visits were conducted at baseline, 12, and 24 months. Participants with biochemical measurements at baseline and at least one follow-up visit were included in the analysis. Informed consent was obtained from participants ≥18 years and from parents of participants <18 years. Informed assent was obtained from participants 13–17 years old. Reasons for non-participation at each stage included personal reasons of study participants, loss to follow-up and time constraints on the part of the participants. The study was approved by the local Institutional Review Board, and it was conducted in accordance with the Health Insurance Portability and Accountability Act. The study was registered on ClinicalTrials.gov (NCT02557438) (date 21 September 2015).

Study Visits: Participants completed study visits after an overnight fast. A detailed medical history was obtained, and a thorough physical examination and anthropometric measurements were performed. Height was obtained using a wall-mounted stadiometer and calculated as the average of three height measurements. Weight was measured to the nearest 0.1 kg using an electronic scale. The formula weight in kg/(height in meters)^2^ was used to calculate BMI. Participants underwent fasting blood tests for glucose, insulin, and glycated hemoglobin (HbA1c), and completed a mixed meal tolerance test (MMTT) following the consumption of a Boost drink. Postprandial glucose and insulin levels were measured at 15, 30, 60, 90, and 120 min during the MMTT. The Boost drink manufacturer changed the dextrose content (50 vs. 75 g per 360 mL of Boost) during the course of the study, thus the specific Boost preparation was used as a covariate in our analyses. Of note, the Boost formulations differed only in dextrose content (50 or 75 gr of dextrose per 350 mL); fat and protein quantity (9 g and 22.5 g, respectively) and quality were identical. Participants completed dual energy x-ray absorptiometry (DXA) and abdominal magnetic resonance imaging (MRI) at each timepoint. Physical activity was measured with the Paffenbarger questionnaire, which collects data on physical activity in hours/week of either moderate or vigorous activity [17]. A 24 h-food recall was administered to each participant by a nutritionist from the Massachusetts General Hospital Clinical and Translational Research Center, and data were analyzed using Nutrition Data System for Research software version 2014 to 2021 depending on year of data acquisition.

Biochemical Analysis: Serum glucose levels were measured using a glucose hexokinase assay (Roche Diagnostics, Indianapolis, IN, USA; intermediate precision (day-to-day) coefficient of variation (CV) of 1.1–1.2%, sensitivity 2 mg/dL) was used to measure serum glucose levels. An ultrasensitive chemiluminescence immunoassay (Beckman Coulter, Fullerton, CA, USA; intra-assay CV 2.0–4.2%, intraassay CV 3.1–5.6%, sensitivity 0.03 uIU/mL) was used to assess serum insulin levels. HbA1c levels were assessed with boronate affinity high performance liquid chromatography (Trinity Biotech, Jamestown, NY, USA; intra- and interassay CV < 2%, sensitivity 3.8%).

Calculation of Glucose Homeostasis Markers: The formula used to calculate Homeostatic Model Assessment for Insulin Resistance (HOMA-IR) was (fasting insulin, uIU/mL) × (fasting glucose, mg/dL)/405. To assess insulin secretion and sensitivity, the insulinogenic index (ISI) was calculated according to the following formulas: ISI-T15: the ratio of change in insulin to change in glucose from 0 to 15 min (∆I15/∆G15); ISI-T30: ratio of change in insulin to change in glucose from 0 to 30 min (∆I30/∆G30) [18]. The oral disposition index (oDI) is a measure of beta cell function adjusted for insulin sensitivity and was calculated as ISI-T30 (∆I30/∆G30) multiplied by 1/fasting insulin level [19,20]. Matsuda index is a marker of insulin sensitivity in hepatic and peripheral tissue and was calculated as (10,000/square root of [fasting glucose × fasting insulin] × [mean glucose × mean insulin during MMTT]) [21,22].

Imaging: Lean mass and fat mass were assessed using DXA (Hologic 4500 A, Waltham, MA, USA). Single-slice abdominal MRI (Siemens Trio; Siemens Medical Systems, Erlangen, Germany) was performed using a T1-weighted fast spin-echo pulse sequence (slice thickness 10 mm; repetition time, 300 ms; echo time, 12 ms; echo train, 4; matrix, 512 × 512) at the level of L4, and cross-sectional areas (cm^2^) of visceral adipose tissue (VAT) and subcutaneous adipose tissue (SAT) were determined with commercial software (VITRAK, Merge/eFilm, Milwaukee, WI, USA) [23].

Statistical Analysis: Stata v.18.0 (StataCorp LP) was used for data analysis. With 24 completers in each group, the study was powered at above 90% at α level 0.05 to detect at least one SD difference in means for the 24-month change in HOMA-IR. Summative data are presented as the mean ± standard error of the mean (SEM) for continuous data, while categorical data are expressed as absolute numbers and percentages. Baseline characteristics were compared using the Student *t*-test or Pearson’s Chi-Squared test (or Fisher’s Exact Test). Within- and between-group changes in clinical characteristics and glucose homeostasis markers over 24 months were compared using linear mixed effects (LMEs) models with a time by group interaction; the formulation of Boost used was included as a covariate in secondary analysis. Given that both males and females were included in the study, we also added sex as a covariate to determine whether our results held after adjusting for sex. For correlations between glucose homeostasis markers and body composition, Spearman’s test was used.

## 3. Results

### 3.1. Baseline Characteristics

Baseline characteristics of study participants are shown in Table 1 and Table 2. Mean age was 18.0 ± 0.3 years. At baseline, one participant in the SG group had T2D with HbA1c of 6.7%, which improved to <5.6% at 24 month follow-up. At baseline, participants undergoing SG had higher weight (*p* = 0.013) and BMI (*p* = 0.003) than participants in the NS group (Table 1). Groups differed for fat mass (*p* = 0.004) and percent fat mass (*p* = 0.008), which were higher in the SG group (Table 2). Percent lean mass was lower in the SG group compared to the NS group (*p* = 0.010). There were no baseline differences between the two groups when comparing glucose homeostasis parameters. Table 3 describes the number of participants on metformin and classification of glycemic status based on the HbA1c level. None of the participants received oral steroids. However, four received inhaled steroids (two in each group). Additionally, two, one in each group, received topical steroids (*p* = 0.380).

### 3.2. Changes in Weight, BMI, and Body Composition

#### 3.2.1. Changes in Weight, BMI, and Body Composition over 12 Months and over 24 Months

**Figure 1:**
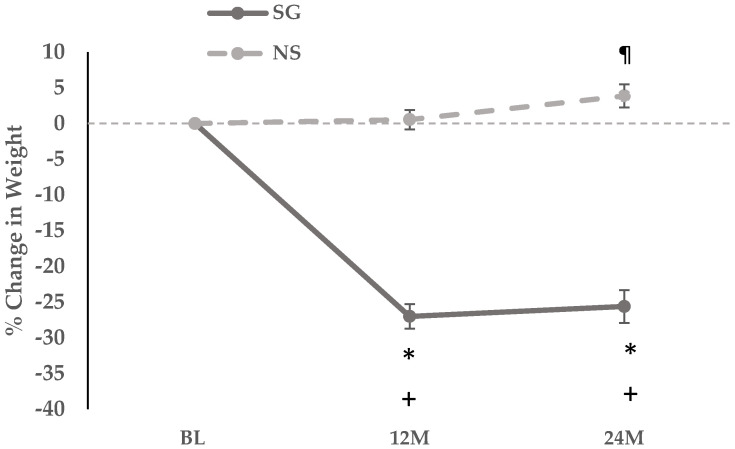
Linear graph showing 12-month and 24-month percent change in weight in the sleeve gastrectomy (surgical) and non-surgical groups. * *p* ≤ 0.05 for between group comparison in longitudinal mixed effects models. + *p* ≤ 0.05 for within-group comparison in longitudinal mixed effects models in the sleeve gastrectomy group. ¶ *p* ≤ 0.05 for within-group comparison in longitudinal mixed effects models in the non-surgical group. Abbreviations: 12 M, 12 months; 24 M, 24 months; BL, baseline; NS, non-surgical group; SG, sleeve gastrectomy group.

The SG group lost 27.1 ± 1.7 percent total body weight over 12 months (*p* < 0.0001), while the NS group gained 0.55 ±1.3 percent total body weight over 12 months (*p* = 0.670). Over 24 months the SG group lost 25.6 ± 2.3 (*p* < 0.0001) while the NS group gained 3.9 ± 1.6 percent total body weight over 24 months (*p* = 0.023) (Figure 1).

Over 12 and 24 months, significant reductions were noted in weight, BMI, BMI z-score, percent excess weight, lean and fat mass, VAT, SAT, and VAT/SAT ratio within the SG group, and across the SG vs. NS groups (*p* < 0.0001) (Table 2).

For body composition, the SG group lost absolute lean mass over 12 and 24 months; in contrast, percent lean mass increased significantly within the group over 12 and 24 months (Table 2). The NS group gained lean mass over 12 and 24 months with an increase in percent lean mass over 12 months, but not over 24 months. The SG group had significant reductions in absolute fat mass over 12 and 24 months, without significant changes within the NS group. Percent fat mass decreased over 12 months in the SG group, with no significant changes noted in the NS group. Similarly, VAT and SAT decreased within the SG group over 12 months and 24 months and increased in the NS group over 24 months. Moreover, the groups differed for changes in the VAT/SAT ratio over 12 and 24 months, with a significant reduction in the VAT/SAT ratio noted within the SG group (but not the NS group) over 12 and 24 months.

Of note, at 12-month and 24- month follow up, most participants in both SG and NS groups were female (12 months: SG, 21 female and six male vs. NS, 24 female and eight male, *p* = 0.062; 24 months: SG, 19 female and four male vs. NS, 22 female and nine male, *p* = 0.979).

#### 3.2.2. Changes in Weight and Body Composition Between 12 and 24 Months

Within the SG group, between 12 and 24 months, there were no significant changes for weight, percent body weight, BMI, BMI z-score, percent excess weight, lean mass, percent lean mass, fat mass, percent fat mass and SAT. However, VAT and the VAT/SAT ratio decreased significantly in the SG group over this period.

Similarly, within the NS group, between 12 and 24 months, there were no significant changes in BMI z-score, lean and fat mass, percent lean and fat mass, VAT, SAT and VAT/SAT ratio. However, total body weight increased by 3.2 ± 1.4 percent (*p* = 0.036) (Figure 1), and a significant increase was also noted in BMI and percent excess weight (Table 2).

Further, between 12 and 24 months, between the SG and NS groups, there were no differences noted in changes in weight, BMI, BMI z-score, percent excess weight, lean and fat mass, percent lean and fat mass, and SAT. However, the groups differed for changes in VAT and VAT/SAT ratio, with a significant decrease noted in both within the SG group (Table 2).

### 3.3. Changes in Glucose Homeostasis Parameters

#### 3.3.1. Within Group Changes in Glucose Homeostasis Parameters over 12 Months and 24 Months

**Figure 2:**
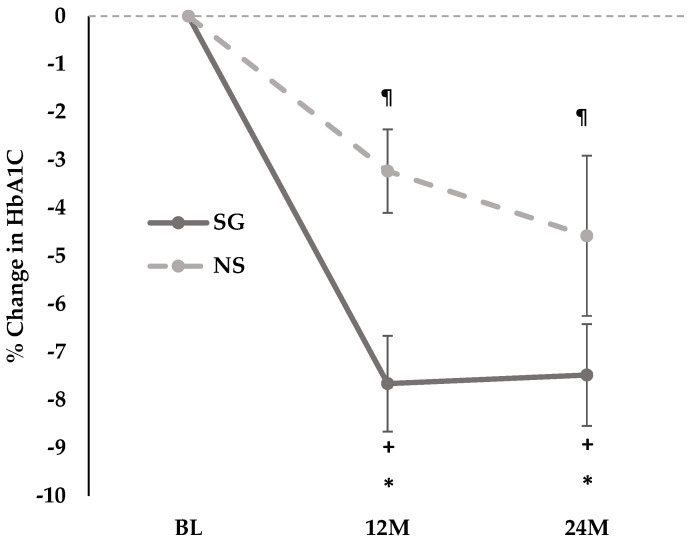
Linear graph showing 12-month and 24-month percent change in HbA1c in the sleeve gastrectomy (surgical) and non-surgical groups. * *p* ≤ 0.05 for between group comparison in longitudinal mixed effects models. + *p* ≤ 0.05 for within-group comparison in longitudinal mixed effects models in the sleeve gastrectomy group. ¶ *p* ≤ 0.05 for within-group comparison in longitudinal mixed effects models in the non-surgical group. Abbreviations: 12M, 12 months; 24M, 24 months; BL, baseline; NS, non-surgical group; SG, sleeve gastrectomy group.

Over 12 and 24 months, within-group reductions in HbA1c were noted in both SG and NS groups, with a greater decrease noted within the SG group (Figure 2, Table 2). Within the SG group, fasting glucose decreased over 24 (but not 12) months, after controlling for type of Boost, while glucose AUC decreased over 12 (but not 24) months, regardless of the type of Boost. No changes in fasting glucose and glucose AUC were noted in the NS group over 12 or 24 months.

Over 12 and 24 months, in the SG group, fasting insulin and HOMA-IR decreased significantly regardless of Boost type. In the NS group, fasting insulin and HOMA-IR increased at 24 months, but this change was not significant after adjusting for Boost type. While ISI-T15 (but not ISI-T30) and the Matsuda index increased significantly in the SG group (regardless of Boost type) at 12 months (no significant change at 24 months), no changes were observed within the NS group for these measures. No significant changes were noted within SG and NS groups for changes over 12 and 24 months in oDI. Of note, ISI and oDI indices reflect insulin secretion relative to insulin sensitivity.

At the 12-month follow-up groups differed for metformin use and for classification by HbA1c in euglycemia, prediabetes and diabetes. However, at the 24-month follow-up, these differences were no longer significant (Table 3).

#### 3.3.2. Between Group Changes in Glucose Homeostasis Parameters over 12 Months and over 24 Months

Over 12 months, groups differed for changes in HbA1c, fasting insulin HOMA-IR and Matsuda index, and these differences persisted over 24 months regardless of Boost type (Table 2). Groups also differed for changes in fasting glucose over 12 and 24 months after controlling for type of Boost. Further, over 24 months, differences were noted between groups for changes in insulin secretion relative to insulin sensitivity, evidenced by between group differences in ISI-T30 and oDI regardless of type of Boost. These results persisted after controlling for sex.

#### 3.3.3. Within and Between Group Changes in Glucose Homeostasis Parameters Between 12 and 24 Months

The decrease in HbA1c occurred mostly during the first year, with no significant difference between the 12- and 24-month follow-up visits (Table 2). Between 12 and 24 months, there were no significant changes in any of the glucose homeostasis parameters in either the SG or NS group. These results persisted after controlling for sex.

#### 3.3.4. Glucose and Insulin Levels During the Mixed Meal Tolerance Test at 0, 12 and 24 Months

**Figure 3:**
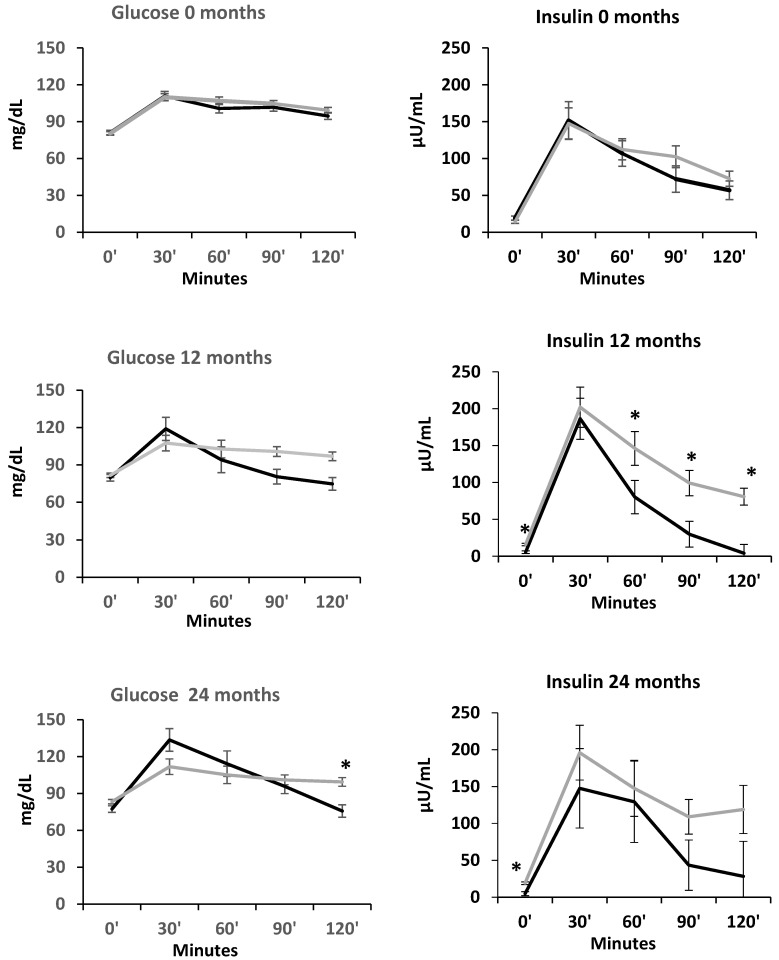
Glucose and insulin levels during the mixed meal tolerance test at 0, 12 and 24 months in participants undergoing sleeve gastrectomy (black lines) versus non-surgical controls (gray lines). * *p* ≤ 0.05 (between groups) adjusting for Boost formulation.

Figure 3 shows the mean glucose and insulin levels in the SG and NS groups at baseline, 12 and 24 months during the MMTT (0, 30, 60, 90 and 120 min timepoints). At 12 and 24 months, fasting insulin levels were lower in SG than NS, and a more rapid decrease in insulin levels was noted in the SG vs. NS group, particularly at 60, 90 and 120 min at the 12-month follow-up visit.

### 3.4. Physical Activity

Groups did not differ for reported physical activity measured by the Paffenbarger questionnaire at baseline, and over 12 and 24 months (Table 4). Groups did differ for changes in physical activity between 12 and 24 months, with a significant increase within the NS group between 12 and 24 months and over 24 months. These findings held after controlling for sex (Table 4).

### 3.5. Nutritional Information

Groups did not differ for reported calorie intake at baseline, over 12 and 24 months, and between 12 and 24 months. Within-group changes over time were also not significant (*p* ≥ 0.183). Similarly, protein intake did not differ across groups at baseline, over 12 months and between 12 and 24 months; changes over 24 months did differ across groups (higher in NS vs. SG groups). Within-group changes over time were not significant (*p* ≥ 0.080). These findings held after controlling for sex (Table 4).

### 3.6. Correlations

#### 3.6.1. Correlations Between 24-Month Changes in Glucose Homeostasis Parameters and 24-Month Changes in BMI and Body Composition

In the whole group, positive correlations were noted between 24-month changes in fasting insulin and HOMA-IR with 24-month changes in BMI, VAT/SAT ratio, and percent fat mass (r ≥ 0.09, *p* ≤ 0.008). Changes in HbA1c correlated positively with changes in BMI and VAT/SAT ratio over 24 months. In contrast, changes in the Matsuda index correlated negatively with changes in BMI, VAT/SAT ratio, and percent fat mass over 24 months in the whole group (r ≥ −0.34, *p* ≤ 0.044).

#### 3.6.2. Correlations Between 12- and 24-Month Changes in Glucose Homeostasis Parameters and 12- to 24-Month Changes in Weight, BMI, and Body Composition

In the whole group, 12- to 24-month changes in fasting insulin correlated positively with 12- to 24-month changes in BMI (r = 0.50, *p* = 0.002). Moreover, in the whole group, 12- to 24-month changes in HOMA-IR correlated positively with 12- to 24-month changes in BMI (r = 0.48, *p* = 0.004) (Table 4). No correlations were found between 12- and 24-month changes in any of the glucose homeostasis parameters and VAT/SAT ratio.

## 4. Discussion

In this study, we observed that over 24 months, groups differed for changes in HbA1c, fasting insulin, HOMA-IR, ISI-T-30, oDI and Matsuda index. Over 24 months, improvements in insulin sensitivity and secretion were observed after SG, with greater decreases in HbA1c compared with the NS group, along with improvements in HOMA-IR and fasting insulin. Our data demonstrate an overall improvement in glucose homeostasis over 24 months following SG, consistent with our hypothesis and prior studies demonstrating high rates of remission of prediabetes and T2D in youth undergoing MBS [5,6,11,24]. In our present study, while these changes in glucose homeostasis parameters were noted over a 24-month period, the changes occurred primarily in the first 12 months following SG with no significant changes noted in any of the glucose homeostasis parameters between or within groups between 12 and 24 months.

Similarly, over 24 months, significant reductions were noted in weight, BMI, VAT, SAT, FM, and LM in the SG vs. NS groups, but only reductions in VAT and VAT/SAT ratio were significant between 12 and 24 months in the SG group. Prior studies in youth and adult populations have similarly demonstrated significant and durable weight and BMI decreases 24 months after MBS, with maximum reductions observed in the initial 12–13 months followed by weight regain in some participants in the subsequent 12 months [6,25].

Associations between changes in glucose homeostasis parameters and changes in BMI or body composition measurements over 24 months and from 12-month follow-up to 24-month follow-up were only found in the group taken as a whole. Over 24 months, positive associations were observed of changes in HOMA-IR and fasting insulin with changes in BMI, VAT/SAT ratio, and percent fat mass. In contrast, negative associations were found between 24-month changes in Matsuda index and 24-month changes in BMI, VAT/SAT ratio, and percent fat mass. Changes over 24 months in HbA1c correlated positively with changes over 24 months in BMI and VAT/SAT ratio. For changes between 12- and 24-month follow-up, positive associations were found of changes in fasting insulin and HOMA-IR with changes in BMI and fat mass. Our results are congruent with prior published data demonstrating that improvements in glucose homeostasis measures tracked with greater weight loss following MBS [26].

In the present study, we also utilized data from the MMTT to closely investigate the effects of SG on insulin resistance and beta cell function over time, along with other measures of glucose homeostasis such as HbA1c, and fasting insulin and glucose. Taken together, we show that SG results in improvements (particularly in insulin resistance parameters) over the first 12 months of surgery, without further improvement over the subsequent 12-month period. These findings are also consistent with our hypothesis, as our participants experienced most of the weight loss and body composition changes in the first 12 months after SG [14,15]. Reductions in BMI, fat mass, and VAT/SAT ratio are correlated with and likely drive improvements in glucose homeostasis parameters, and the stabilization of these measures between 12 and 24 months likely explains the lack of further significant changes in measures of glucose homeostasis in the second year following SG. A prior study in adults similarly showed significant improvements in glycemic measures during the active weight loss phase in the first 12 months following MBS, which were then sustained during the weight stability phase between 12 and 36 months after MBS [27]. While active weight loss appears to be the main contributor to the enhancements in glycemic control, studies have implicated other physiological changes following MBS that appear to have an effect on glucose homeostasis physiology, such as changes in incretins, gut microbiota, bile acids, and glucose transporters [28,29,30,31,32,33,34]. Additional investigations are needed to closely examine the long-term progression of these physiological alterations following MBS. However, as other authors have described, early weight loss may lead to better long term weight maintenance [35]. Thus, early intervention with weight loss medications when weight starts to plateau could aid in achieving more favorable BMI curves over time, and consequently, sustain favorable glucose homeostasis outcomes [35].

Interestingly, over 24 months and between the 12- and 24-month follow-up visits the NS group increased their physical activity, and over 12 and 24 months, lean mass in the NS group increased, while that in the SG group decreased. In contrast, percent lean mass increased in both SG and NS groups over 12 months, and in the SG group over 24 months. However, groups did not differ for changes in lean mass or percent lean in the interval between 12- and 24-month follow-up. The lack of lean mass increase in the SG group over this period may have contributed to the glucose homeostasis plateau observed in our study. A continued increase in lean mass may allow for continued improvement of glucose homeostasis parameters after weight changes plateau following surgery [36].

It is important to note that compared to prior studies, our cohort at baseline had a narrower glycemic range with only one participant having pre-existing T2D. Nevertheless, our data support the fact that SG is an effective treatment option for metabolic complications of obesity; however, medical therapies, such as glucagon-like peptide 1 (GLP-1) agonists, may need to be introduced after a year of SG in order to achieve further improvements in glucose homeostasis if clinically warranted.

Our study has its limitations. Firstly, at baseline, not all participants had complete MMTT data. Second, there was a decrease in the number of participants at each follow-up visit along with a decrease in the number of participants with complete data for glucose homeostasis parameters, which could have reduced the statistical power of the study and increased the risk of bias. Third, due to the nature of our study, participants were not randomized to each group (SG vs. NS); thus, our results could have been influenced by pre-existing differences between the groups other than the intervention. Fourth, we were not powered to determine correlations between glucose homeostasis parameters and determinants of these parameters within each group; larger studies are necessary to conclusively determine factors that predict changes in glucose homeostasis parameters following surgery, as well as causal relationships. Finally, our results may not be generalizable, as the predominant sex was female, with few male participants (although controlling for sex did not alter our results), and our cohort mostly included euglycemic and prediabetic participants, with an insufficient representation of youth with diabetes.

## 5. Conclusions

In conclusion, in this study we demonstrate an improvement in glucose homeostasis parameters 24 months after surgery. However, it is important to note that these changes occur mainly during the first year after surgery, without significant changes between the first and the second year. Medical management may be necessary when weight starts to plateau following SG for continued and sustained improvements in glucose homeostasis parameters.

## Figures and Tables

**Table 1:** Baseline characteristics for sleeve gastrectomy (surgical) and non-surgical groups.

	Surgical Group *n* = 28	Non-Surgical Group *n* = 37	*p*-Value ^a^
**Age (years)**	18.2 ± 0.4	17.9 ± 0.5	0.604
**Sex (Female/Male)**	22/6	27/10	0.773
**Height (cm)**	168.0 ± 1.8	166.8 ± 1.3	0.580
**Weight (kg)**	133.9 ± 5.0	119.1 ± 3.4	**0.013**
**BMI (kg/m^2^)**	47.3 ± 1.3	42.6 ± 0.9	**0.003**
**BMI z-score**	2.6 ± 0.1	2.5 ± 0.1	0.176
**Excess Body Weight (kg)**	71.1 ± 4.3	58.5 ± 2.8	**0.013**
**% Excess Body Weight**	87.9 ± 7.3	77.2 ± 7.0	0.302

Data expressed as absolute number or as mean ± SEM. Abbreviations: BMI, body mass index. ^a^ *p* for between-group comparison using the Student *t*-test or Pearson’s Chi-Squared. test. *p* values ≤ 0.05 are bolded.

**Table 2:** Baseline and changes over 24 months in anthropometric measurements, body composition, and glucose homeostasis parameters for sleeve gastrectomy (surgical) and non-surgical groups.

	Baseline SG *n* = 28 NS *n* = 37	*p*-Value ^a^	12 Months SG *n* = 27 NS *n* = 32	Change–over 12 Months (12-Month-Baseline)	*p*-Value ^b^	24 Months SG *n* = 23 NS *n* = 31	Change over 24 Months (24-Month-Baseline)	*p*-Value ^b^	Change Between 12 and 24 Months (24–12 Months) SG *n* = 23 NS *n* = 26	*p*-Value ^b^
**Weight (kg)**										
SG	133.9 ± 5.0	**0.013**	98.7 ± 4.5 ^c^	−35.1 ± 2.6 (*n* = 28)	**<0.0001**	99.9 ± 4.7 ^c^	−33.0 ± 3.4 (*n* = 23)	**<0.0001**	0.6 ± 1.8 (*n* = 22)	0.246
NS	119.1 ± 3.4		119.6 ± 4.0 (*n* = 31)	0.6 ± 1.5 (*n* = 31)		123.1 ± 4.2 ^c^ (*n* = 30)	4.4 ± 1.8 (*n* = 30)		3.5 ± 1.7 ^c^	
**BMI (kg/m^2^)**										
SG	47.3 ± 1.3	**0.003**	34.4 ± 1.3 ^c^	−12.8 ± 0.9 (*n* = 28)	**<0.0001**	34.9 ± 1.4 ^c^	−11.9 ± 1.1 (*n* = 23)	**<0.0001**	0.2 ± 0.6 (*n* = 22)	0.175
NS	42.6 ± 0.9		42.3 ± 1.2 (*n* = 31)	−0.3 ± 0.6 (*n* = 31)		43.7 ± 1.2	1.2 ± 0.6 (*n* = 30)		1.4 ± 0.6 ^c^	
**BMI z-score**										
SG	2.6 ± 0.1	0.176	1.9 ± 0.1 ^c^	−0.7 ± 0.1	**<0.0001**	1.8 ± 0.1 ^c^	−0.7 ± 0.1 (*n* = 22)	**<0.0001**	−0.03 ± 0.1 (*n* = 21)	0.489
NS	2.5 ± 0.1		2.4 ± 0.1	−0.1 ± 0.03		2.4 ± 0.1	−0.04 ± 0.04		0.01 ± 0.03	
**% Excess Weight Loss**										
SG	NA	NA	−53.2 ± 3.0 ^c^	NA	**<0.0001**	−51.4 ± 3.2 ^c^	NA	**<0.0001**	1.0 ± 2.6 (*n* = 22)	0.169
NS	NA		1.4 ± 2.7 (*n* = 31)	NA		8.5 ± 2.8 ^c^ (*n* = 30)	NA		6.6 ± 3.2 ^c^	
**Lean Mass (kg)**										
SG	64.0 ± 2.0 (*n* = 27)	0.436	54.5 ± 2.1 ^c^ (*n* = 27)	−9.9 ± 0.9 (*n* = 27)	**<0.0001**	56.0 ± 2.2 ^c^ (*n* = 22)	−7.4 ± 1.5 (*n* = 21)	**<0.0001**	1.2 ± 1.6 (*n* = 20)	0.965
NS	61.7 ± 1.8 (*n* = 35)		63.5 ± 1.8 ^c^ (*n* = 30)	1.9 ± 0.6 (*n* = 28)		64.2 ± 1.9 ^c^	2.3 ± 1.0 (*n* = 29)		0.8 ± 1.2 (*n* = 24)	
**% Lean Mass**										
SG	49.1 ± 0.8 (*n* = 27)	**0.010**	57.9 ± 1.2 ^c^ (*n* = 27)	9.1 ± 1.1 (*n* = 27)	**<0.0001**	59.4 ± 2.0 ^c^ (*n* = 22)	10.2 ± 2.6 (*n* = 21)	**<0.0001**	1.8 ± 2.7 (*n* = 20)	0.500
NS	52.1 ± 0.8 (*n* = 35)		53.3 ± 1.1 ^c^ (*n* = 29)	1.4 ± 0.6 (*n* = 27)		52.4 ± 1.8 (*n* = 28)	0.5 ± 0.8 (*n* = 27)		−0.99 ± 0.9 (*n* = 23)	
**Fat Mass (kg)**										
SG	66.4 ± 2.6 (*n* = 26)	**0.004**	39.9 ± 2.4 ^c^ (n = 27)	−27.1 ± 2.1 (*n* = 26)	**<0.0001**	43.1 ± 2.8 ^c^ (*n* = 22)	−21.8 ± 3.4 (*n* = 20)	**<0.0001**	2.7 ± 2.4 (*n* = 20)	0.748
NS	56.1 ± 2.0 (*n* = 35)		55.4 ± 2.2 (*n* = 30)	−0.8 ± 1.3 (*n* = 28)		57.4 ± 2.4	1.1 ± 2.1 (*n* = 29)		2.7 ± 2.4 (*n* = 24)	
**% Fat Mass**										
SG	50.4 ± 0.8 (*n* = 26)	**0.008**	41.6 ± 1.1 ^c^ (*n* = 26)	−9.4 ± 1.1 (*n* = 26)	**<0.0001**	45.9 ± 1.9 (*n* = 22)	−5.1 ± 3.0 (*n* = 20)	0.221	3.7 ± 3.0 (*n* = 20)	0.111
NS	47.0 ± 0.8 (*n* = 35)		46.1 ± 1.1 (*n* = 29)	−1.0 ± 0.6 (*n* = 27)		46.0 ± 1.8 (*n* = 28)	−0.7 ± 1.3 (*n* = 27)		0.42 ± 1.6 (*n* = 23)	
**VAT (cm^2^)**										
SG	121.1 ± 12.4 (*n* = 25)	0.582	62.6 ± 10.5 ^c^ (*n* = 25)	−59.7 ± 7.3 (*n* = 24)	**<0.0001**	56.1 ± 11.1 ^c^ (*n* = 22)	−59.5 ± 7.9 (*n* = 20)	**<0.0001**	−9.0 ± 3.6 ^c^ (*n* = 19)	**0.034**
NS	108.2 ± 10.6 (*n* = 34)		118.3 ± 9.2 (*n* = 28)	13.3 ± 6.5 (*n* = 27)		122.7 ± 9.7 ^c^ (*n* = 26)	12.4 ± 6.2 (*n* = 24)		7.6 ± 6.0 (*n* = 19)	
**SAT (cm^2^)**										
SG	748.0 ± 31.1 (*n* = 25)	0.108	461.8 ± 31.9 ^c^ (*n* = 25)	−297.0 ± 32.7 (*n* = 24)	**<0.0001**	475.3 ± 35.5 **^c^** (*n* = 22)	−277.9 ± 35.9 (*n* = 20)	**<0.0001**	5.9 ± 23 (*n* = 19)	0.890
NS	673.8 ± 26.6 (*n* = 34)		695.2 ± 28.2 (*n* = 28)	23.0 ± 15.9 (*n* = 27)		714.5 ± 31.1 **^c^** (*n* = 26)	42.9 ± 19.5 (*n* = 24)		23.9 ± 17.5 (*n* = 19)	
**VAT/SAT Ratio**										
SG	0.16 ± 0.02 (*n* = 25)	0.905	0.14 ± 0.01 ^c^ (*n* = 25)	−0.03 ± 0.01 (*n* = 24)	**0.006**	0.11 ± 0.01 **^c^** (*n* = 22)	−0.05 ± 0.02 (*n* = 20)	**<0.0001**	−0.03 ± 0.01 ^c^ (*n* = 19)	**0.012**
NS	0.16 ± 0.01 (*n* = 34)		0.17 ± 0.01 (*n* = 28)	0.01 ± 0.01 (*n* = 27)		0.18 ± 0.01 (*n* = 26)	0.01 ± 0.01 (*n* = 24)		0.01 ± 0.001 (*n* = 19)	
**HbA1c (%)**										
SG	5.6 ± 0.1 (*n* = 25)	0.382	5.1 ± 0.1 ^c,e^ (*n* = 27)	−0.6 ± 0.1 (*n* = 26)	**0.002 ^d^**	5.1 ± 0.1 ^c,e^ (*n* = 20)	−0.4 ± 0.1 (*n* = 18)	**0.029**	−0.02 ± 0.05 (*n* = 19)	0.985
NS	5.5 ± 0.7 (*n* = 24)		5.3 ± 0.1 ^c,e^ (*n* = 29)	−0.2 ± 0.1 (*n* = 17)		5.3 ± 0.1 ^c,e^ (*n* = 30)	−0.3 ± 0.1 (*n* = 21)		−0.01 ± 0.1 (*n* = 23)	
**Fasting Glucose (mg/dL)**										
SG	81.3 ± 1.5 (*n* = 28)	0.947	79.0 ± 1.4 (*n* = 27)	−3.1 ± 1.5 (*n* = 28)	0.058 ^d^	79.5 ± 1.7 ^e^ (*n* = 19)	−1.6 ± 1.8 (*n* = 19)	0.405 ^d^	0.3 ± 1.0 (*n* = 18)	0.244
NS	82.2 ± 1.5 (*n* = 37)		83.0 ± 1.3 (*n* = 29)	1.1 ± 1.6 (*n* = 29)		81.4 ± 1.5 (*n* = 29)	0.3 ± 1.4 (*n* = 29)		−2.2 ± 1.0 (*n* = 22)	
Glucose AUC (mg/dL * 2 h)										
SG	12,244 ± 306 (*n* = 23)	0.655	11,197 ± 316 ^c,e^ (*n* = 23)	−928 ± 337 (*n* = 19)	0.083	12,139 ± 495 (*n* = 10)	563 ± 400 (*n* = 8)	0.743	−2 ± 687 (*n* = 7)	0.606
NS	12,828 ± 277 (*n* = 34)		12,067 ± 296 (*n* = 25)	81 ± 251 (*n* = 23)		12,440 ± 411 (*n* = 17)	242 ± 382 (*n* = 16)		269 ± 371 (*n* = 12)	
**Fasting Insulin (uIU/mL)**										
SG	18.6 ± 2.7 (*n* = 28)	0.133	7.2 ± 1.6 ^c,e^ (*n* = 27)	−13.5 ± 2.8 (*n* = 27)	**<0.0001 ^d^**	7.5 ± 2.1 ^c,e^ (*n* = 20)	−14.5 ± 3.9 (*n* = 20)	**<0.0001 ^d^**	0.7 ± 1.0 (*n* = 19)	0.395
NS	14.2 ± 2.7 (*n* = 35)		16.4 ± 1.5 (*n* = 32)	3.0 ± 2.1 (*n* = 30)		19.0 ± 2.0 ^c^ (*n* = 27)	3.8 ± 2.4 (*n* = 25)		2.3 ± 2.4 (*n* = 22)	
**HOMA-IR**										
SG	3.7 ± 0.5 (*n* = 28)	0.170	1.4 ± 0.3 ^c,e^ (*n* = 27)	−2.7 ± 0.6 (*n* = 27)	**<0.0001 ^d^**	1.5 ± 0.5 ^c,e^ (*n* = 19)	−3.2 ± 0.9 (*n* = 18)	**<0.0001 ^d^**	0.12 ± 0.2 (*n* = 17)	0.580
NS	2.9 ± 0.6 (*n* = 35)		3.5 ± 0.3 (*n* = 29)	0.6 ± 0.5 (*n* = 27)		4.0 ± 0.4 ^c^ (*n* = 25)	0.9 ± 0.5 (*n* = 24)		−0.12 ± 0.5 (*n* = 18)	
**ISI-T15**										
SG	2.4 ± 0.8 (*n* = 23)	0.151	4.2 ± 0.7 ^c,e^ (*n* = 26)	0.8 ± 0.4 (*n* = 22)	0.573	3.7 ± 1.0 (*n* = 11)	0.4 ± 1.1 (*n* = 8)	0.879	0.49 ± 0.5 (*n* = 9)	0.567
NS	3.9 ± 0.8 (*n* = 31)		4.1 ± 0.7 (*n* = 26)	0.8 ± 1.1 (*n* = 23)		4.9 ± 0.8 (*n* = 17)	1.0 ± 1.6 (*n* = 15)		0.67 ± 1.1 (*n* = 13)	
**ISI-T30**										
SG	5.1 ± 1.4 (*n* = 23)	0.705	6.7 ± 2.4 (*n* = 26)	1.1 ± 2.6 (*n* = 22)	0.567	3.3 ± 1.4 (*n* = 11)	−3.2 ± 1.7 (*n* = 8)	**0.034 ^d^**	0.23 ± 1.0 (*n* = 9)	0.806
NS	6.3 ± 1.3 (*n* = 32)		9.3 ± 1.1 (*n* = 25)	2.7 ± 2.3 (*n* = 23)		6.2 ± 1.1 (*n* = 17)	−0.3 ± 1.1 (*n* = 16)		−1.0 ± 2.7 (*n* = 12)	
**oDI**										
SG	0.7 ± 0.2 (*n* = 23)	0.739	2.0 ± 1.1 (*n* = 26)	1.3 ± 1.5 (*n* = 22)	0.218	1.2 ± 0.4 (*n* = 11)	0.9 ± 0.9 (*n* = 8)	**0.044 ^d^**	0.87 ± 0.7 (*n* = 9)	0.843
NS	0.6 ± 0.2 (*n* = 32)		0.5 ± 1.1 (*n* = 25)	−0.02 ± 0.3 (n = 23)		0.6 ± 0.3 (*n* = 17)	−0.1 ± 0.1 (*n* = 16)		−0.15 ± 0.2 (*n* = 12)	
**Matsuda Index**										
SG	4.8 ± 0.7 (*n* = 22)	0.343	8.7 ± 1.0 ^c,e^ (*n* = 23)	5.7 ± 1.1 (*n* = 18)	**<0.0001 ^d^**	9.2 ± 1.3 (*n* = 11)	6.9 ± 3.3 (*n* = 8)	**0.002 ^d^**	2.7 ± 4.5 (*n* = 7)	0.873
NS	4.4 ± 0.7 (*n* = 31)		3.1 ± 1.0 (*n* = 24)	−1.8 ± 0.8 (*n* = 22)		3.8 ± 1.0 (*n* = 17)	−1.3 ± 0.8 (*n* = 15)		−0.3 ± 0.6 (*n* = 12)	

Data expressed as marginal mean ± SEM. Abbreviations: HbA1c, glycated hemoglobin; AUC, area under the curve; BMI, body mass index; HOMA-IR, Homeostatic Model Assessment for Insulin Resistance; ISI, insulinogenic index; NS, non-surgical group; oDI, oral disposition index; SAT, subcutaneous adipose tissue; SG, surgical group; VAT, visceral adipose tissue. T15: 15 min after administration of the mixed meal; T30: 30 min after administration of the mixed meal. Number of participants who had assessment of a specific variable are indicated in each cell if the number differed from the number of participants indicated in the column header. ^a^ *p* for between-group comparison using the Student *t*-test. ^b^ *p* for between-group comparison in longitudinal mixed models. *p* values ≤ 0.05 for between-group comparisons using the Student *t*-test and between-group comparison in longitudinal mixed models are bolded. ^c^ *p* ≤ 0.05 for within-group comparison in longitudinal mixed models. ^d^ *p* ≤0.05 for between-group comparison in longitudinal mixed models adjusting for Boost formulation consumed during mixed meal tolerance test. ^e^ *p* ≤ 0.05 for within-group comparison in longitudinal mixed models adjusting for Boost formulation consumed during mixed meal tolerance test. * Over 2 hours after Boost consumption during the mixed meal tolerance test (MMTT).

**Table 3:** Metformin use and clinical classification by glycated hemoglobin at baseline, 12 months, and 24 months in sleeve gastrectomy and non-surgical groups (absolute numbers).

	Baseline		*p*-Value	12 Months		*p*-Value	24 Months		*p*-Value
	SG	NS		SG	NS		SG	NS	
**Metformin Use**									
Yes	5	7	0.612	0	8	**0.003 ^a^**	1	5	0.138
No	20	20		25	19		21	22	
Unknown	3	10		2	5		1	3	
**HbA1c Classification**									
Euglycemia < 5.7%	18	17	0.101	27	24	**0.008 ^a^**	20	26	0.159
Prediabetes 5.7–6.4%	6	7		0	5		0	4	
Diabetes > 6.4%	1	0		0	0		0	0	

Data expressed as absolute numbers. Abbreviations: HbA1c, glycated hemoglobin; NS, non-surgical group; SG, sleeve gastrectomy group. ^a^ *p* ≤ 0.05 for between-group comparison using Pearson’s Chi-Squared test.

**Table 4:** Changes over 24 months in physical activity as assessed by the Paffenbarger questionnaire and in nutritional intake as assessed by 24 h food recall.

	Baseline SG *n* = 28 NS *n* = 37	*p*- Value ^a^	12 Months SG *n* = 27 NS *n* = 32	Change over 12 Months (12 Months-Baseline)	*p*- Value ^b^	24 Months SG *n* = 23 NS *n* = 31	Change over 24 Months (24 Months-Baseline)	*p*- Value ^b^	Change Between 12 and 24 Months (24–12 Months) SG *n* = 23 NS *n* = 26	*p*- Value ^b^
**Physical Activity** **(h/week)**										
SG	28.8 ± 4.2 (*n* = 26)	0.951	39.1 ± 3.8 (*n* = 25)	8.5 ± 4.9 **^c^** (*n* = 23)	0.103	31.1 ± 4.3 (*n* = 22)	5.8 ± 4.7 (*n* = 21)	0.188	−7.8 ± 4.9 (*n* = 19)	**0.002**
NS	28.8 ± 3.5 (*n* = 37)		28.2 ± 3.3 (*n* = 32)	0.61 ± 4.9 (*n* = 32)		40.3 ± 3.8 (*n* = 28)	13.9 ± 4.7 **^c^** (*n* = 28)		9.4 ± 5.1 **^c^** (*n* = 23)	
**24 h Caloric** **Intake**										
SG	1404 ± 157 (*n* = 27)	0.183	1173 ± 123 (*n* = 25)	−317.7 ± 157.8 (*n* = 24)	0.320	1395 ± 181 (*n* = 22)	4.9 ± 230.8 (*n* = 22)	0.372	333.1 ± 142.3 (*n* = 19)	0.881
NS	1669 ± 134 (*n* = 37)		1675 ± 109 (*n* = 32)	−73.3 ± 181.1 (*n* = 32)		1907 ± 155 (*n* = 30)	139.0 ± 182.6 (*n* = 30)		271.8 ± 240.2 (*n* = 25)	
**24 h Protein** **Intake (grams)**										
SG	76.0 ± 7.9 (*n* = 28)	0.800	61.1 ± 6.9 (*n* = 26)	−18.6 ± 7.8 (*n* = 26)	0.215	61.0 ± 8.8 (*n* = 22)	−12.3 ± 10.5 (*n* = 23)	**0.030**	1.19 ± 6.5 (*n* = 22)	0.199
NS	73.5 ± 6.2 (*n* = 37)		73.8 ± 6.2 (*n* = 32)	−3.4 ± 9.5 (*n* = 32)		88.8 ± 7.7 (*n* = 30)	10.3 ± 9.2 (*n* = 31)		13.6 ± 9.7 (*n* = 26)	

Data expressed as marginal mean ± SEM. ^a^ *p* for between-group comparison using the Student *t*-test. ^b^ *p* for between-group comparison in longitudinal mixed models. ^c^ *p* ≤ 0.05 for within-group comparison in longitudinal mixed models. *p* values ≤ 0.05 for between-group comparisons using the Student *t*-test and between-group comparison in longitudinal mixed models are bolded.

## Data Availability

Datasets generated and analyzed during the current study are not publicly available but are available from the corresponding author on reasonable request.

## Abbreviations

The following abbreviations are used in this manuscript:

MBS	Metabolic and bariatric surgery
SG	Sleeve gastrectomy
NS	Non-surgical
BMI	Body mass index
HOMA-IR	Homeostatic Model Assessment for Insulin Resistance
T2D	Type 2 diabetes mellitus
RYGB	Roux-en-Y gastric bypass
IR	Insulin resistance
BCF	Betha cell function
MMTT	Mixed meal tolerance test
DXA	Dual energy x-ray absorptiometry
CV	Coefficient variation
ISI	Insulinogenic index
oDI	Oral disposition index
VAT	Visceral adipose tissue
SAT	Subcutaneous adipose tissue
HbA1c	Glycated hemoglobin
FM	Fat mass
LM	Lean mass
GLP-1	Glucagon-like peptide 1
AUC	Area under the curve
